# Recent trends in airway management

**DOI:** 10.12688/f1000research.10311.1

**Published:** 2017-02-17

**Authors:** Joelle Karlik, Michael Aziz

**Affiliations:** 1 Oregon Health & Science University, Portland, OR, USA

**Keywords:** tracheal intubation, video laryngoscopy, surgical airway, apneic oxygenation, awake intubation

## Abstract

Tracheal intubation remains a life-saving procedure that is typically not difficult for experienced providers in routine conditions. Unfortunately, difficult intubation remains challenging to predict and intubation conditions may make the event life threatening. Recent technological advances aim to further improve the ease, speed, safety, and success of intubation but have not been fully investigated. Video laryngoscopy, though proven effective in the difficult airway, may result in different intubation success rates in various settings and in different providers’ hands. The rescue surgical airway remains a rarely used but critical skill, and research continues to investigate optimal techniques. This review highlights some of the new thoughts and research on these important topics.

## Introduction

Airway management remains a difficult skill to master, requiring hands-on training with human patients and extensive clinical experience. Fortunately, most intubations are not challenging in experienced hands. Current prediction models to anticipate the difficult intubation are poor and have limited application to new tools other than the traditional direct laryngoscopy (DL). Recent reviews of cannot-intubate cannot-oxygenate (CICO) events have continued to highlight the low rate of success for rescue despite the application by experienced anesthesiologists and surgeons ^1^. Fortunately, recent advances in airway management have facilitated improved intubation conditions by augmenting oxygenation during laryngoscopy and providing indirect video laryngeal views. This review will briefly discuss recent research in these areas, highlighting that all devices may not be appropriate in all health-care settings. Further emphasis is placed on recent research and recommendations on the definitive surgical airway in the CICO scenario.

## Apneic oxygenation

Apneic oxygenation is not a new concept, but new research has begun to emphasize its potential value during airway management. Oxygenation via standard nasal cannula can be limited by patient comfort and maximum flow rates. A recent study demonstrated that high-flow nasal cannula at 15 mL/min does not increase time to desaturation in the critically ill population ^2^. These populations with increased oxygen consumption and large shunts may require higher delivery of apneic oxygenation than standard nasal cannula can provide.

Alternative devices offer higher concentrations of inspired oxygenation as well as increased comfort, including humidification. Various techniques for this enhanced apneic oxygenation include buccal RAE (Ring-Adair-Elwyn) tubes ^3^, transnasal humidified rapid-insufflation ventilatory exchange (THRIVE) devices ^4, 5^, and dual-use laryngoscopes ^6^. These techniques show potential to substantially prolong apnea time. The THRIVE was recently found to be as effective as face-mask pre-oxygenation at maintaining oxygenation during rapid sequence induction ^7^.

## Video laryngoscopy

Video laryngoscopy (VL) has been shown to increase the rate of first-attempt success in patients with the predicted difficult airway in operating rooms ^8– 10^. Accordingly, recent literature has focused on extending the use of VL to various settings, including pre-hospital, emergency department, and intensive care unit (ICU). These settings often present a high-stress scenario with an actively decompensating patient with widely varying provider experience. Current data suggest that the success seen with VL may not automatically translate to other settings outside the operating room.

### The pre-hospital setting

VL has clear benefits and disadvantages in the pre-hospital setting. VL may have a special role for intubations in the field where patients are challenged with spine immobilization precautions, suboptimal positioning, and altered facial anatomy or trauma. However, secretions, blood, and vomitus may obscure VL cameras, and camera lenses are difficult to clear of contamination. In addition, first responders may have different training and comfort levels with new devices.

The Airtraq (Prodol, Vizcaya, Spain), C-MAC (Karl Storz, Tuttlingen, Germany), and GlideScope (Verathon, Burnaby, BC, Canada) devices have been directly compared with DL in the pre-hospital emergency intubation setting. Initial observational studies have shown an association with decreased number of intubation attempts when using a GlideScope versus DL ^11^. A retrospective study in a pre-hospital paramedic helicopter system observed increased first-pass success and a decrease in overall attempts with C-MAC laryngoscopy ^12^. A prospective observational study of out-of-hospital intubations showed improved first-pass success with rapid sequence intubations but did not significantly change overall first-pass success when compared with DL ^13^.

Further randomized research has questioned these benefits. Two prospective randomized trials of pre-hospital VL observed decreased rates for first-pass success when compared with DL ^14, 15^. In another study, VL was significantly more successful in patients with a grade II–IV DL view, underlying the importance and success of indirect VL in patients with difficult DL views ^16^.

VL may have a unique role in the pre-hospital setting where certain patient and environmental factors limit an adequate DL view ^17^. In contrast, pre-hospital practitioners have a wide variety of training, skill, and experience that may limit their comfort with new devices. Given the challenges seen with indirect VL in the pre-hospital setting, we believe that devices with both DL and VL capabilities may be best in these environments, but this hypothesis is currently untested in the literature.

### The emergency department setting

VL has also been studied in the emergency department setting where experience and a controlled hospital setting differ from pre-hospital emergency airways. Observational studies have demonstrated higher first-attempt success rates with VL over DL in the emergency department ^18, 19^. However, a prospective randomized study showed no significant increase in first-pass success with C-MAC versus DL blades when used by senior residents in emergency resident training ^20^. A similar study of more experienced anesthesia providers showed no difference in first-pass success despite improved glottic views ^21^. Overall, VL may not change initial intubation success in the emergency department but may be beneficial for subsequent attempts, but prospective randomized data are limited.

### The intensive care setting

The intensive care setting often presents urgent or emergent airways in an actively decompensating patient. Observational data demonstrate a higher intubation success rate for VL over DL ^22, 23^. Furthermore, in prospective randomized trials, the rate of successful first intubation was significantly higher with GlideScope VL than with DL in the hands of fellow-trained physicians, a finding also found in a study of resident physicians ^24, 25^. However, a different trial with fellow physicians showed no significant differences in first-pass intubation success comparing a variety of VL and DL devices ^26^. A recent multicenter study including the McGrath MAC video laryngoscope (Medtronic, Minneapolis, MN, USA) versus Macintosh laryngoscope (MACMAN) trial clarified optimal laryngoscopy technique in the ICU setting, but results again suggest that there is no difference in success rate between VL and DL in the ICU environment ^27^. The utility of VL in this environment, like that in other emergency environments, may be limited by the presence of blood in the airway ^28^.

### Cardiopulmonary resuscitation

Intubation during cardiopulmonary resuscitation (CPR) provides definitive airway control and essential information about ongoing resuscitation via expired carbon dioxide. Unfortunately, successful intubation is often hampered by patient movement due to chest compressions. During CPR events, there was no difference in successful intubations or speed of intubation when prospectively comparing the GlideScope VL with DL ^29^. However, interruptions in chest compressions were minimized with VL in two different studies ^29, 30^. In addition, VL was noted to have higher success rates when used by novice physicians during ongoing CPR ^30^.

### Awake intubation

Awake intubations present a high-stress challenge with an anticipated difficult airway with a need for smooth, comfortable, and rapid intubation. In contrast to the scenarios discussed above, awake intubations are typically performed by providers with specific training in awake airway management techniques. Flexible bronchoscopic techniques are traditionally used for awake intubations because of their flexibility and comfort, but recent studies have investigated VL as an alternative technique.

A randomized trial of oral awake intubations with the flexible bronchoscope versus McGrath Series 5 VL (Teleflex, Morrisville, NC, USA) found no change in time to intubation or overall intubation success ^31^. Average patient comfort scores after topical and regional anesthesia and sedation were similar for the two techniques. However, a high dropout rate in the VL group due to failed regional transtracheal block may limit interpretation of this study. Another randomized trial compared flexible bronchoscopic intubation with C-MAC D-blade VL for nasal awake intubations under topical anesthesia. It also showed no difference in the success of intubations ^32^. These findings were validated in healthy volunteers in the awake upright (sitting) position ^33, 34^. GlideScope VL was compared with fiber-optic techniques for the awake intubation of morbidly obese patients, a population with a higher rate of difficulty airway, with similar rates of successful intubation on first attempt ^35^. These cumulative findings suggest that VL may be a useful alternative to flexible bronchoscopic intubation in select patient populations with anticipated difficult airways.

## Surgical airway

### Scalpel versus cannula

The CICO scenario requiring surgical airway is a rare and stressful occurrence. Despite ongoing research and education in this area, the overall success of anesthesiologist-placed emergency surgical airway appears to be very low. The 4th National Audit Project (NAP4) recorded surgical airway management across the United Kingdom during a 1-year period. Most surgical airways were performed by a surgeon with 100% success ^1^. In contrast, only 9 out of 25 (36%) anesthesiologists were successful in rescuing the airway with a surgical technique. Review of these cases underlined the importance of a simple rapid technique with an optimal chance of success in unpracticed and stressed providers.

Guidelines are currently divided between two techniques: scalpel or cannula airways. The 2015 Difficult Airway Society guidelines strongly favor scalpel techniques over cannula techniques, a change from the 2004 guidelines ^36^. In contrast, cannula techniques such as percutaneous cricothyrotomies are still included in similar guidelines, including the Australian and New Zealand College of Anaesthetists (ANZCA) Guidelines on Equipment to Manage a Difficult Airway During Anaesthesia ^37^.

Scalpel airways are thought to be faster, simpler, and overall more successful than cannula techniques. In the NAP4, 100% of first-choice surgical cricothyrotomies (3 out of 3) were successful, but the low number of first-choice scalpel airways may limit study interpretation ^1^. An observational study of success rates of trauma intubation and airway rescue techniques in both anesthesiologists and other physicians showed a 100% success rate of traditional scalpel approaches ^38^. However, the overall rate of surgical airways was lower than previously reported. These studies, though limited by their observational approach and low denominators, add to a growing amount of evidence that surgical cricothyrotomies may have a higher success rate when compared with cannula techniques ^39, 40^.

Advocates of cannula techniques note that the needle-wire movements are more familiar to anesthesiologists, but cannula techniques may be vulnerable to a high rate of complications in the CICO scenario. The NAP4 analysis found that 90% of cricothyrotomies (26 out of 29) were first attempted with cannula techniques, potentially reflecting provider preference. However, 58% of cannula cricothyrotomies (15 out of 26) performed by anesthesiologists failed and required rescue ^1^. The cannula may provide a conduit for oxygenation via jet ventilation to temporize airway management, but barotrauma remains a significant risk in the obstructed airway. A systematic review of emergency transtracheal jet ventilation, including 90 out of 132 CICO scenarios (68%), showed higher rates of device failure, barotrauma, and overall complications in CICO emergency scenarios when compared with non-CICO emergencies ^41^.

### Different scalpel techniques

There is a lack of randomized data supporting any scalpel technique ^36^. Many techniques have similar steps (neck extension, identification of the cricothyroid membrane, incision through the skin and cricothyroid membrane, and insertion of a cuffed tracheal tube) with variations including the number of incisions and methods to keep the incision open. The 2015 Difficult Airway Society guidelines recommend a scalpel-bougie technique for tracheal cannulation with a gum-elastic bougie to keep the incision open ^16, 36^. Other techniques, including the “4-step technique”, include devices such as tracheal hooks that may not be immediately available ^42^. Training models in humans and animal cadaveric models have not favored one technique over another ^42– 44^.

Given the challenges in surgical airway training, recent studies have investigated which techniques have higher success in both inexperienced and experienced learners. A recent study in surgical airway-naïve trainees showed a higher rate of success when using scalpel techniques versus cannula techniques on cadavers ^45^. Recent studies of needle versus scalpel techniques in animal cadaveric models show that senior anesthesiologists also achieve a higher rate of success with surgical scalpel techniques ^46^. However, more research is required before any definitive conclusions can be made about ease of learning with the different techniques.

### Use of ultrasound

One of the more recent innovations in airway management is ultrasonography for the identification of landmarks for the surgical airway. The thought is that airway structures can be identified before induction of anesthesia so that emergency surgical airway management is performed with sound knowledge of the underlying airway anatomy. The growing number of obese patients significantly increases the likelihood of a difficult-to-assess surface anatomy. In addition, patients with previous head-and-neck trauma, surgery, or radiation (or a combination of these) may have difficult or impossible anatomy to identify by surface structure.

Patient selection for ultrasound identification of airway structures impacts its utility. A study of emergency medicine physicians and senior residents showed no significant difference in the ability to identify the cricothyroid membrane with palpation or ultrasound in healthy volunteers ^47^. In contrast, a study of women in labor showed that only 39% of anesthesiologists correctly identified the cricothyroid membrane in obese patients compared with 71% of non-obese patients ^48^. A study of overweight volunteers also showed a significant increase in ability to identify the cricothyroid membrane with ultrasound versus palpation (100% to 46%, respectively) ^49^. A study in human cadavers showed that naïve trainees were over five times more successful in cannulating the trachea with ultrasound guidance than palpation in difficult or impossible anatomy ^50^. So it appears that ultrasound is a useful tool for the identification of surgical airway structures in the obese patient population or others with difficult-to-identify surface airway structures.

Given the low incidence of CICO airways, most providers should be considered novice regardless of years in practice. Despite the known benefits of both cognitive and procedural skills training in the CICO scenario, there are few standardized training programs. The current US Accreditation Council for Graduate Medical Education (ACGME) anesthesiology requirements do not require programs to train graduates in the surgical airway ^51^. The ANZCA provides standards for CICO training sessions but does not clarify which algorithm is preferred ^52^. In addition, such training programs are not often used by anesthesiologists or others who manage the airway, regardless of practice settings.

## Future directions

As laryngoscopy devices continue to evolve, the literature continues to investigate whether these new devices improve intubation success and patient outcomes. Currently, initial data suggest that new techniques such as VL are not universally superior to traditional DL. Variations in setting, patient characteristics, situational stress, and provider training likely contribute to overall device success and are confounders that cannot all be modified.

A recent focus on CICO events and surgical airway management has highlighted the importance of simple, rapid techniques with ongoing training throughout one’s career. Ultrasonography provides a helpful adjunct for the identification of surgical airway access anatomy when surface landmarks are unclear and should be introduced in surgical airway training.

Regardless of the technique, quick decision-making, familiarity with equipment, proper training, and effective communication remain the most important factors in a successful airway intervention. Fortunately, these characteristics can be improved with educational interventions and simulation training. As intubation techniques, devices, and evidence grow, airway education must continue to do so as well. The greatest opportunity for airway success remains in a straightforward plan with familiar equipment and practiced techniques.
